# Identification of Protein Carbonyls (PCOs) in Canine Serum by Western Blot Technique and Preliminary Evaluation of PCO Concentration in Dogs With Systemic Inflammation

**DOI:** 10.3389/fvets.2020.566402

**Published:** 2020-12-09

**Authors:** Beatrice Ruggerone, Graziano Colombo, Saverio Paltrinieri

**Affiliations:** ^1^Department of Veterinary Medicine, University of Milan, Milan, Italy; ^2^Veterinary Teaching Hospital, University of Milan, Lodi, Italy; ^3^Department of Biosciences, University of Milan, Milan, Italy

**Keywords:** CRP–C-reactive protein, dog, oxidative stress, PON-1, paraoxonase 1, sepsis

## Abstract

In people, serum Protein Carbonyls (PCOs) increase during oxidative stress (OS) due to oxidative damage to proteins. OS is often associated with inflammation and especially with sepsis, a condition hard to diagnose in veterinary medicine because reliable markers are lacking. The aim of this study was to assess whether PCOs in canine serum may be detected by antibody-based methods such as Western Blotting (WB), and to preliminarily investigate the possible utility of this marker in dogs with inflammation. A serum sample oxidized *in vitro* was used to set up the method; the coefficient of variation obtained by repeated analysis varied from 24 to 36%. In order to assess whether the technique may cover the range of PCOs concentration detectable in routine practice, PCOs were measured in 4 healthy dogs and in 15 with inflammatory diseases, in some cases potentially associated with sepsis, as suggested by the results of other inflammatory markers such as C-Reactive Protein (CRP) and the anti-oxidant enzyme Paraoxonase 1 (PON-1): the concentration of PCOs was low in dogs with normal PON-1 activity, moderately increased in the majority of dogs with low-normal PON-1 activity, and severely increased in dogs with very low PON-1 activity. In conclusion this study demonstrates that PCOs, may be detected in canine serum, using antibody-based techniques such as WB. The preliminary results in dogs with and without systemic inflammation encourage further studies on the possible role of PCOs as inflammatory markers.

## Introduction

The generation of reactive oxygen species (ROS) occurs in a variety of physiological and pathological conditions. The presence of ROS leads to protein oxidation; in particular ROS cause protein side chains oxidation, with the subsequent generation of protein carbonyl (PCO) groups (aldehydes and ketones) (1). The amount of PCOs in blood increases under pathological conditions related to oxidative stress. In people, protein carbonylation is the most widely used biomarker for oxidative damage to proteins, since it reflects cellular damage induced by multiple forms of ROS (2).

Inflammation is characterized by oxidative phenomena and the detection of protein carbonyls (PCOs) in biological samples may be used to quantify the level of oxidative stress (OS) (3) associated with inflammation. It is expected that, in septic patients, OS is higher than in subjects with inflammation not associated with sepsis (4), and therefore PCOs may be markers of sepsis. For example, in people, the concentration of plasma protein carbonyl is significantly higher in septic patients compared with controls (4).

There are many methods used nowadays for the evaluation of the plasmatic concentration of PCOs. Among these, the most employed is based on derivatization of proteins using 2,4-dinitrophenylhydrazine (DNPH) (3, 5); DNPH reacts with PCO and leads to the formation of 2,4-dinitrophenylhydrazone (DNP), a stable compound that can be detected and quantified through different methods (5). With the discovery of antibodies able to recognize the DNP adducts, it has been possible to increase the sensitivity of PCO detection. Antibody-based methods have been developed to investigate the concentration of PCOs. These methods include dot blot, immunochemistry, ELISA and Western blot (WB) (6–10). In veterinary medicine, the diagnosis of sepsis is based on the direct detection of pathogens and/or on the “indirect” evaluation of the effects exerted by the pathogens on the organism (increased temperature, heart and respiratory rate, changes in the leukogram, altered serum concentration of acute phase proteins, modified serum protein electrophoresis); however, the parameters currently used to support a clinical diagnosis of sepsis are not enough sensitive and specific. To the best of our knowledge, in veterinary medicine, few studies have been developed about carbonyls, mainly to focus about OS and inflammatory conditions related to pregnancy in healthy dogs (11), associated with a diet rich in polyunsaturated fatty acids (12) or in diabetes mellitus in dogs (13) and in cats (14). Different substrates and techniques have been used to quantify PCOs and rarely a significant difference from sick and healthy animals was found, as in the study of Zini et al. (14) that demonstrated an increased concentration of PCOs in the erythrocyte membranes of diabetic cats compared with control cats. No studies about inflammation and PCOs levels in dogs is nowadays reported.

Of consequence, considering the importance of PCOs in human medicine, it would be useful to assess whether PCOs could be a good marker of inflammation and/or sepsis also in dogs. As a first step of this approach, information on the analytical performances of reliable methods to detect PCOs are needed. Therefore, the aims of our study were to assess if a sensitive method such as Western blotting (WB) is able to detect PCO in canine serum and to investigate, using this method, the actual presence of PCO in canine serum. In order to preliminarily assess whether the test may measure the concentrations of PCOs detectable in routine practice, the method was applied also on sera from dogs with inflammatory diseases, on which PCOs are expected to increase.

## Method

### Samples and Study Design

The first part of the study was focused on the establishment of the procedure. To this aim, 400 μg of serum from one healthy dog were oxidized “*in vitro*” with 10% (v/v) cigarette smoke extract (CSE) for 1 h at room temperature. The CSE-treated serum was separated using Micro Bio-Spin™ P-6 Gel Columns (Biorad, Hercules, California, USA) to eliminate residual CSE, and control and CSE-treated samples were subjected to WB analysis as described below.

Moreover, to assess the repeatability of the method, 7 naïve (i.e., non CSE-treated) serum samples randomly selected from dogs (two healthy dogs as control, three dogs with pyometra, one dog with pyelonephritis and one with systemic parasitic disorder) were analyzed in 6 consecutive working days using the WB method described below. The coefficient of variation (CV) was then established, based on the mean value and the standard deviation of the 5 working sessions using the formula: CV = SD/Mean × 100.

For the third and retrospective part of this study, four serum samples from healthy dogs and fifteen serum samples collected at the Veterinary teaching hospital of the University of Milan from privately owned dogs with diseases potentially associated with inflammation (pyelonephritis, pyometra, pneumonia, discospondylitis, parvovirus, and systemic parasitic disease), including inflammatory conditions with a possible septic base, and stored at the Department of Veterinary Medicine, were analyzed.

Dogs were considered healthy or sick on the basis of physical examination, routine biochemistry and hematology, ultrasound or radiographic examination when necessary. Whole blood was collected from the jugular or the cephalic vein, placed in tubes without anti-coagulant and centrifuged within 2 h of collection to obtain serum that has been frozen at −20°C until analyzed. EDTA samples were analyzed within 2 h of collection. Samples were collected during routine veterinary visits for health monitoring and an owner's written consent that allows the use of residual amount of samples for research purposes has been signed for every dog. Therefore, based on the regulation of the Ethical Committee of our Institution (Decision n° 2/2016) a formal approval of the Ethical Committee is not needed.

### Western Blot Analysis

The procedure was described by Levine et al. (5). Two-hundreds μL of 10 mM 2,4-dinitrophenylhydrazine (DNPH) in HCl 2N, also known as Brady's reagent, were added to 1 mL of 1 mg/mL of serum protein solution. A similar amount of carbonylated HSA (0.22 nmol PCO/mg protein) was used as standard. DNPH molecule reacts with carbonyl groups leading to the formation of the stable 2,4 dinitrophenylhydrazone (DNP). A blank sample with 200 μL of 2N HCl (without DNPH) with 1 mL of protein sample was also prepared. Each tube was incubated in the dark at room temperature for 1 h and briefly vortexed every 15 min during the incubation. Tubes were added with 1.2 mL of 20% trichloroacetic acid (TCA), placed on ice for 15 min, and centrifuged at 10.000 × g for 10 min at 4°C in a microcentrifuge. The supernatant was then removed and the pellet resuspended in 1 mL of (1:1) ethanol/ethyl acetate mixture then vortexed in order to remove any free DNPH. These two latter passages were repeated until supernatants was completely transparent. After the final wash, the protein pellets were resuspended in 500 μL of Laemmli Sample Buffer and incubated at 90°C for 5–10 min. After cooling at room temperature, samples were centrifuged 5 min at 10.000 × g and supernatants were transferred to a new tube. After protein quantification with the Bradford assay, protein samples labeled with 2,4-dinitrophenylhydrazine (3 μg) were run on SDS–PAGE on Tris–HCl 10% resolving gels and electroblotted onto an Immobilon P polyvinylidene difluoride (PVDF; Sigma-Aldrich, Milan, Italy) membrane and stored at −20°C for later use. A two-steps immunodetection protocol employing primary 1:40,000 anti-DNP antibodies (rabbit IgG fraction (cod. A6430) from Molecular Probes (Eugene, OR, USA) and 1:80.000 horseradish peroxidase (HRP)-conjugated secondary antibodies [goat anti-rabbit IgG (cod. G21234), from Molecular Probes] was performed as described in Colombo et al. (3). The signal was developed with Enhanced Chemilumionescence (ECL) using ChemiDoc™ Touch Imaging System (Bio-Rad Laboratories, Hercules, California, USA) and PVDF membranes were stained with Amido Black. Chemiluminescent PCO levels and Amido Black images were densitometrically quantified using a specific software (Image Lab™ Software, Bio-Rad, Hercules, California, USA).

To quantify the protein carbonyl content of dog sera, we performed a detailed optimization of the required protein amount for the analysis. This is necessary in order to ensure that both Amido Black signals and ECL signals are ideal for a correct quantification. To allow protein quantification on PVDF membrane after Amido Black staining, we used a preliminary calibration line generated by loading 1, 2, 3, 4, 5, 6, 7, 8, 9, and 10 micrograms of dog serum proteins in each lane of the electrophoresis gel. After separation, proteins were transferred to PVDF membrane and stained with Amido Black. After ChemiDoc™ Touch Imaging System scanning and Image Lab™ Software quantification of all the entire lanes, we observed that 6 micrograms of proteins are sufficient to generate a saturated signal. For this reason, we used a second calibration line loading 0.5, 1, 1.5, 2, 2.5, 3, 3.5, 4, 4.5, and 5 micrograms of dog serum proteins in each lane. After Amido Black staining, ChemiDoc™ Touch Imaging System scanning and Image Lab™ Software quantification of all the entire lanes, we observed that the range 0.5–5 micrograms of proteins is ideal to generate a linear and useful signals. To allow protein carbonyl quantification of ECL signals, we used the “accumulation mode” of ChemiDoc™ Touch Imaging System. This mode allowed us to generate a digital image of the ECL signal each 10 s of exposition. In parallel, the software is able to highlight whether the signal of each single lane is saturated or optimal for quantification. After optimization of Amido Black and ECL quantification, we used Image Lab™ Software to quantify both signals for each dog serum sample and we expressed the ratio between ECL and Amido Black intensity in arbitrary units. More specifically, the ratio between the carbonyl signal intensity and the protein signal intensity defined the relative protein carbonyl content (3). Results are expressed as arbitrary units (A.U.) considering the mean value of controls as reference.

### Clinical Chemistry

To better focus on the inflammatory and oxidative status of the dogs included in this study, we evaluated the serum concentration of C-reactive protein (CRP), the major acute phase protein of dogs (15) and the serum activity of the antioxidant enzyme paraoxonase 1 (PON-1), whose metabolism is strictly associated with that of both PON-1 and CRP (16). These biochemical analyses were performed using an automated chemistry analyzer (Cobas Mira; Roche Diagnostics, Basel, Switzerland) as previously described (17, 18).

### Statistical Analysis

Results of PCO, CRP and PON-1 were correlated to each other using a Spearman correlation test run in an excel-based specific software (Analyse-it v 4.90, Analyse-it software Ltd, Leeds, UK).

## Results

### Detection of PCO in Canine Serum

**Figure 1:**
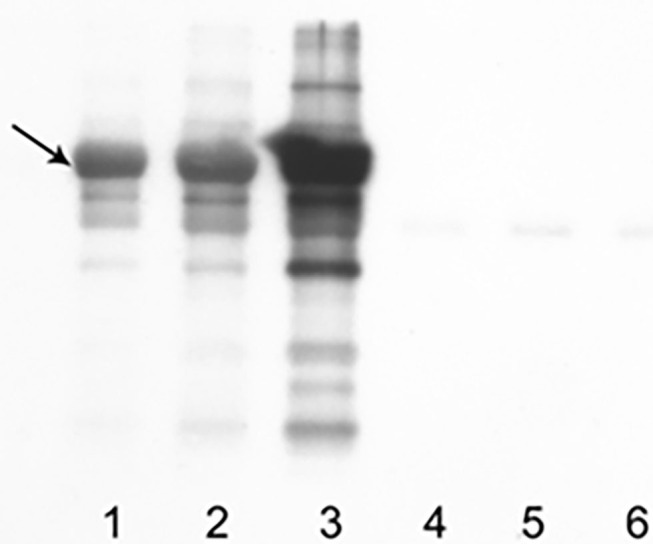
Results obtained with WB on control samples and samples oxidized with CSE. A band of apparent MW of 69 kDa (black arrow), consistent with carbonylated dog serum albumin, is overt. In lanes 1–3, 10 μg of sample added with DNPH were loaded (1: control, 2: solution with oxidized serum at 5%, 3: solution with oxidized serum at 10%); in lanes 4–6, same quantity and dilution of sera but without DNPH.

Western blot showed an evident band of apparent molecular weight of 69 kDa, consistent with carbonylated dog serum albumin (Figure 1). The intensity of the band was weak in naïve serum but strong in the serum oxidized with CSE and even stronger when the percentage of oxidized serum increases from 5 to 10%. In serum oxidized with CSE, the concentration of PCO was 2.02 nmol/mg (Figure 1). This value was then used as a positive control in the further analysis of results obtained in healthy and sick dogs. The coefficient of variation of the 6 sequential reading varied from 24 to 36% (median CV = 30%).

### Results Recorded in Healthy and Sick Dogs

**Figure 2:**
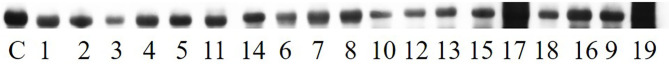
Results obtained with WB on samples collected from healthy dogs (lanes 1–4) and from clinically sick dogs (5–19). The control sample (serum oxidized with CSE) has been loaded on lane C. Dog serum proteins has been separated in SDS-PAGE, transferred to PVDF membrane and immunodetected with specific antibodies.

Results from healthy and sick dogs are reported in Table 1 and Figure 2, respectively.

**Table 1:** Values of PON-1, CRP, and PCO in the examined samples.

**Dog n**°****	**Diagnosis**	**PCO (A.U.)**	**CRP n.v. <10.4** **(mg/L)**	**PON n.v. > 116** **(U/mL)**	**SIRS** **score**
1	Healthy	1.17	Nd	Nd	Nd
2	Healthy	0.96	Nd	Nd	Nd
3	Healthy	0.82	Nd	Nd	Nd
4	Healthy	1.04	Nd	Nd	Nd
5	Pyelonephritis	2.23	162.0	106.6	Positive
6	Pyelonephritis	1.63	315.1	113.4	Na
7	Pyelonephritis	1.32	194.9	101.5	Na
8	Pyelonephritis	2.16	39.6	113.5	Na
9	Pyelonephritis	2.92	5.6	125.3	Na
10	Angiostrongylus	0.98	100.0	147.7	Positive
11	Pyometra	2.66	195.4	114.9	Na
12	Pyometra	0.94	78.0	110	Na
13	Pyometra	0.71	10.7	112.7	Na
14	Pyometra	2.24	1186.3	103.5	Positive
15	Pneumonia	0.98	395.0	160.8	Positive
16	Pneumonia	2.14	173.3	119.6	Positive
17	Parvovirus	33.56	39.3	43.1	Positive
18	Parvovirus	1.48	182.9	119.6	Positive
19	Discospondylitis	49.65	96.3	75.3	Positive

PCO, Protein carbonyl; CRP, C-reactive protein; PON, Paraoxonase 1; A.U., Arbitrary Unit; n.v., normal value; Nd, Not determined.

*Number 13 and 14 are referred to the same dog before and after therapy*.

All the sick dogs had clinical or laboratory signs or diagnostic imaging findings consistent with inflammation (i.e., fever, pain and/or discharges, and/or leukocytosis). The presence of systemic inflammation was supported also by CRP values, that in the sick group were higher than the reference intervals either in terms of mean and median values (211.6 ± 290.9 mg/L; 162.0 mg/L) or in terms of results of individual dogs, since and all but two (dog n° 9 and 13) had CRP values higher than the upper reference limit. Systemic inflammation was also supported by the presence of a positive SIRS score (19) in all the cases on which the available clinical information allowed the calculation of the score (Table 1). Similarly, mean and median PON-1 values were lower than the lower reference limit (111.2 ± 27.1 U/mL; 113.4 U/mL) and, with rare exceptions (dogs n° 9, 10, 15) PON-1 values of individual dogs were low-normal or lower than the lower limit of the reference interval established in a previous study (17). In 2 cases (dogs n° 17 and 19) PON-1 activity was largely lower than the lower limit of the reference interval.

The concentration of PCOs in healthy dogs corresponded to 1.00 ± 0.15 A.U (median: 1.00 A.U.). Although this study was not focused on the statistical comparison of results of healthy and sick dogs, the analysis of results recorded in sick dogs revealed a higher mean and median concentration of PCOs (7.04 ± 14.4 A.U. and 2.14 A.U. respectively), although the variability among tested sera was very high. More specifically, the concentration of PCOs was low and comparable with that of healthy dogs in 2 out of 3 dogs with normal PON-1 activity and increased in one of these dog, that had both CRP and PON-1 activity within the reference interval. Moreover, PCOs were moderately increased, with values consistent with those recorded in the positive control (canine serum oxidized with CSE) in the large majority of dogs with low-normal PON-1 activity, and severely increased in the two dogs with very low PON-1 activity. Both these dogs had diseases possibly associated with a septic condition, such as parvovirosis, and discospondylitis, which is known to originate from hematogenous spread of bacteria or fungi from distant foci of infection (20, 21). However, in sick dogs, PCOs and PON-1 were not correlated to each other (*P* = 0.091; *r* = −0.356). Conversely, the increase of CRP was not apparently associated with changes in PCOs: the CRP value of the two dogs with very high PCOs was only moderately increased, and, on the contrary, PCOs values in dogs with high or very high CRP concentration were low or moderately increased. Also in this case no correlations were found between the two analytes (*P* = 0.737; *r* = −0.095).

## Discussion

This study evidenced that antibody-based techniques such as WB may detect protein carbonyls in canine serum over a broad range of concentrations, likely associated with the presence of systemic inflammation, and the comparison of results regarding PCOs and PON-1 suggests that this association likely depends on oxidations that may occur in severe inflammatory conditions.

The discovery of reliable markers of oxidation had a two-fold-benefit: an early antibiotic administration is determinant for the survival of patients with severe inflammation or sepsis (22) and, on the other hand, the overuse of broad-spectrum antibiotics in patients with a false-positive diagnosis of sepsis can generate drug-resistant pathogens.

Since a clear marker of sepsis is lacking in veterinary medicine, we designed this study to assess whether PCOs may be used to support the suspicion of an oxidative pathogenesis in dogs with inflammation as a preliminary step toward future studies specifically related to septic inflammation. The first step of this study was to assess whether antibody-based techniques, that have been shown to be able to react with DNP adducts in other species (3), may work in dogs. To this aim, we employed a western blot technique already used in other species (2). With this method, we confirmed the presence of PCOs in oxidized canine serum. The most evident protein band evidenced by this method in the dog sera was considered as represented by albumin because of its MW (69 kDa) and because of its abundance. Moreover, we showed that the positive signal increases with the percentage of CSE, suggesting a good linearity of the method. However, the repeatability of the method is not excellent, since the CV is higher than those recommended for most of the analytes currently employed in veterinary medicine (23).

The application of this method to canine sera from dogs with inflammation, demonstrated that increases of PCOs may occur independently on the magnitude of changes in CRP concentration. Conversely, increases of PCOs seems to be inversely associated with decreases of PON-1 activity. The lack of a statistical correlation may depend on the low number of observations, on the possible presence of oxidations not associated with inflammation (as suggested by one single dog with normal CRP and increased PCOs) or on the wide individual variability of the response of both the analytes. Despite the lack of statistical significance, results of PCOs and PON-1 seem to be inversely proportional to each other in most dogs: increases of PCOs were moderate, but higher than the intrinsic variability of the method demonstrated by repeatability assays, and consistent with values obtained after experimental oxidation of sera with CSE, in dogs with low-normal PON-1 activity and severe in dogs with very low PON-1 activity. In turn, changes in PON-1 activity were not correlated in magnitude or frequency with changes of CRP but this confirms what already demonstrated in other studies (17) and likely depends on the different magnitude of oxidative phenomena associated with inflammation between different patients. In other words, severe decreases of PON-1 activity may be found only in those dogs with inflammation on which oxidative stress is particularly severe. From this standpoint, the similar behavior of PCOs and PON-1 suggests that both the markers identified oxidations only in some of the dogs with systemic inflammation included in this study.

This was a preliminary study focused on the analytical validation of the western blot approach, on which sera from dogs enrolled during routine veterinary clinical activities were included only to draw provisional information on the possible practical application of this technique in healthy dogs and in dogs with inflammation, irrespective of the final diagnosis of sepsis, in order to examine serum samples that are expected to have different concentration of PCOs. Therefore, the caseload included also a low number of dogs on which the diagnosis of sepsis was presumed on the basis of the final diagnosis and/or on the presence of changes in inflammatory markers of inflammation such as CRP and/or PON-1. Sepsis was not confirmed in these dogs and therefore it was not possible to differentiate dogs with septic SIRS and dogs with SIRS associated with local infection. However, it is possible that the huge inter-individual variability in the concentration of PCOs may actually depend on the presence/absence of sepsis. Hence, based on these results, it would be advisable to perform larger case-control studies, with a better clinical evaluation of sepsis. In veterinary medicine, the presence of sepsis could be suspected based on the presence of infectious agents in animal on which systemic inflammation is detected through clinical or laboratory data that concur to achieve the SIRS score (e.g., heart and respiratory rate, temperature, and WBC/μL) (19). Due to the retrospective nature of the current study, it was not possible to collect this information in all the dogs included in the study but, in future studies on the clinical utility of PCOs, it would be necessary to better standardize patient's enrolment through the SIRS score. Moreover, it would be interesting to perform longitudinal studies based on sequential samplings during the follow up, in order to investigate the possible prognostic role of PCOs based on the outcome of the disease. Another potential limitation of this study is that Western blotting, although still considered the gold standard in many laboratory settings, is time consuming and operator-dependent. This may be a limitation for the application on a large scale of this method to support a clinical diagnosis of SIRS or sepsis associated with oxidative stress. Other methods that may allow to process larger batches of samples such as the spectrophotometric evaluation of protein-DNP adducts characterized by a peak absorbance at 366 nm, or the application of antibody-based methods to detect DNP adducts on ELISA plates or on automated immunoturbidimetric systems, would improve the practical applicability of PCO measurement in routine practice.

In conclusion, we demonstrated that antibody-based methods to detect DNP adducts associated with protein oxidation may identify the presence of carbonylated proteins in canine serum. Moreover, through a preliminary analysis of samples from dogs with inflammation potentially associated with oxidative stress, we provided evidence that increases of PCOs are evident in dogs in which the activity of the antioxidant enzyme PON-1 is decreased, thus supporting the possible role of PCOs, eventually associated with PON-1, in differentiating dogs with oxidative stress associated with SIRS from those with systemic or localized inflammation in which oxidative stress plays a minor role. Further studies on a larger caseload of dogs enrolled with a more standardized clinical approach, and possibly using simplest antibody-based methods are needed to confirm the possible diagnostic or prognostic role of PCOs as a marker of inflammation or sepsis in dogs.

## Data Availability Statement

The original contributions presented in the study are included in the article/supplementary materials, further inquiries can be directed to the corresponding author/s.

## Ethics Statement

Samples were collected during routine veterinary visits for health monitoring and an owner's written consent that allows the use of residual amount of samples for research purposes has been signed for every dog. Therefore, based on the regulation of the Ethical Committee of the University of Milan (Decision n° 2/2016) a formal approval of the Ethical Committee is not needed.

## Author Contributions

BR collected the samples and drafted the manuscript. GC performed the experiments and supervised the results. SP conceived of the presented idea, supervised the findings of this work, and drafted the manuscript. All authors contributed to the design of the study and analysis, interpretation of data, also critically revised the manuscript, read, and approved the final version.

## Conflict of Interest

The authors declare that the research was conducted in the absence of any commercial or financial relationships that could be construed as a potential conflict of interest.
